# The cortical oscillatory patterns associated with varying levels of reward during an effortful vigilance task

**DOI:** 10.1007/s00221-020-05825-8

**Published:** 2020-06-07

**Authors:** Adam Byrne, Katerina Kokmotou, Hannah Roberts, Vicente Soto, John Tyson-Carr, Danielle Hewitt, Timo Giesbrecht, Andrej Stancak

**Affiliations:** 1 Department of Psychological Sciences, University of Liverpool, Liverpool, L69 7ZA UK; 2 Institute for Risk and Uncertainty, University of Liverpool, Liverpool, UK; 3 Unilever Research and Development, Port Sunlight, UK; 4 Centre for Social and Cognitive Neuroscience (CSCN), School of Psychology, Universidad Adolfo Ibáñez, Santiago, Chile

**Keywords:** Event-related desynchronization, Value, Discounting, Cognitive effort

## Abstract

We explored how reward and value of effort shapes performance in a sustained vigilance, reaction time (RT) task. It was posited that reward and value would hasten RTs and increase cognitive effort by boosting activation in the sensorimotor cortex and inhibition in the frontal cortex, similar to the horse-race model of motor actions. Participants performed a series of speeded responses while expecting differing monetary rewards (0 pence (p), 1 p, and 10 p) if they responded faster than their median RT. Amplitudes of cortical alpha, beta, and theta oscillations were analysed using the event-related desynchronization method. In experiment 1 (*N* = 29, with 12 females), reward was consistent within block, while in experiment 2 (*N* = 17, with 12 females), reward amount was displayed before each trial. Each experiment evaluated the baseline amplitude of cortical oscillations differently. The value of effort was evaluated using a cognitive effort discounting task (COGED). In both experiments, RTs decreased significantly with higher rewards. Reward level sharpened the increased amplitudes of beta oscillations during fast responses in experiment 1. In experiment 2, reward decreased the amplitudes of beta oscillations in the ipsilateral sensorimotor cortex. Individual effort values did not significantly correlate with oscillatory changes in either experiment. Results suggest that reward level and response speed interacted with the task- and baseline-dependent patterns of cortical inhibition in the frontal cortex and with activation in the sensorimotor cortex during the period of motor preparation in a sustained vigilance task. However, neither the shortening of RT with increasing reward nor the value of effort correlated with oscillatory changes. This implies that amplitudes of cortical oscillations may shape upcoming motor responses but do not translate higher-order motivational factors into motor performance.

## Introduction

Cognitive effort is prevalent in a number of settings such as education (Von Stumm et al. 2011; Cacioppo et al. 1996), the workplace (Kidwell Jr and Bennett 1993; Van Iddekinge et al. 2018), and consumer behaviour (Heidig et al. 2017). In psychiatric or mood disorders (e.g., depression), a loss of motivation to face cognitively or physically challenging tasks has been reported (Treadway et al. 2012; Cohen et al. 2001). However, while the decision to make an effort has been extensively researched, and the subjective experience of effort is familiar to most people, the effects of reward and the value of effort on performance in an effortful task and the neural basis of this are not yet fully understood.

In behavioural economic theories of decision making, effort is framed as a discounting factor that reduces the value of rewards when an effort is required to achieve them (Inzlicht et al. 2014; Kurzban et al. 2013). The discounting effect of effort can be measured using the COGED method (Westbrook et al. 2013; Westbrook and Braver 2015), which offers staircase iterated rewards across multiple levels of effort until an indifference point is reached, indicating the amount of money required for participants to agree to put more effort into the task (Westbrook et al. 2013; Massar et al. 2016). The value of effort, determined using COGED, has been shown to correlate with individual engagement (Westbrook et al. 2013) and performance (Massar et al. 2016) in cognitive tasks. Further, the level of engagement in a cognitive task can be manipulated by varying performance-based rewards (Massar et al. 2016; Dinges and Powell 1985; Knutson et al. 2000).

The discounting effect of cognitive effort has been attributed to a number of processes (Gailliot and Baumeister 2007; Lazarus 1993; Tooby and Cosmides 2008; Christie and Schrater 2015), but is commonly thought to be the consequence of top-down cognitive control (Botvinick and Braver 2015; Kaplan and Berman 2010; Shenhav et al. 2013). This would be required to control task-relevant cortical activation and inhibition at the expense of task-irrelevant activation and inhibition, and may be localised to the dorsal anterior cingulate cortex, which has been implied to mediate cognitive control during attentional tasks (Shenhav et al. 2013).

Processes which may to be controlled during motor actions are proposed by the horse-race theory of motor inhibition in the stop-signal task (Logan and Cowan 1984; Band et al. 2003; Schultz 2015). This model posits opposing processes of motor readiness during stop-signal tasks, where motor activation occurs in response to a ‘GO’ signal and motor inhibition occurs in response to a ‘STOP’ signal, and a movement is only successfully inhibited if the inhibitive processes complete before the movement is finished, meaning that successful responses to ‘STOP’ signals are based on the relative speed of these competing processes (for more information see Band et al. 2003, Fig. 1).

Visual acuity (Mathewson et al. 2009), visual detection threshold (Ergenoglu et al. 2004), visual discrimination (Hanslmayr et al. 2005) and pain sensitivity (Babiloni et al. 2006) have been shown to be enhanced if stimuli occur during a period of suppressed alpha-band oscillations. In a similar vein, motor readiness or preparation seconds before a self-paced voluntary movement (Chatrian et al. 1959), or during an imagined, or observed movement (Nagai and Tanaka 2019; Pfurtscheller et al. 2005), often manifests in amplitude decreases of cortical alpha- and beta-band oscillations (Pfurtscheller and Berghold, 1989; Tzagarakis et al. 2010, 2015; Fox et al. 2016; Ishii et al. 2019). This has been found to increase prior to self-paced finger movements requiring large force (Stancak et al. 1997), and during fast compared to slow movements (Stancak and Pfurtscheller 1996a, b). Suppressions of alpha- and beta-band power may, therefore, be representative of the excitatory processes posited by the horse-race theory.

Conversely, inhibitory processes are employed in tasks which require withholding a response under the state of strong motor readiness, for example during a stop-signal task (Leimkuhler and Mesulam 1985). Cortical inhibition or idling has been found to manifest as an increase in the amplitude of alpha- or beta-band oscillations (Visani et al. 2019; Korzhik et al. 2018; Salmelin and Hari 1994; Pfurtscheller et al. 1996; Jensen et al. 2005; Fry et al. 2016), and frontal beta-band synchronisation has been shown to occur during periods of motor inhibition (Alegre et al. 2006; Wessel and Aron 2013; Swann et al. 2009; Fonken et al. 2016; Wagner et al. 2018). Functional brain imaging studies point to a major role of the right prefrontal cortex in employing the inhibition of motor actions (Feng et al. 2014; Garavan et al. 2002; Simmonds et al. 2008), perhaps through dopaminergic innervations (Miller and D'Esposito 2005; Fuster 2015; Chao and Knight 1995). Moreover, frontal beta-band synchronisation has been shown to occur during periods of motor inhibition (Alegre et al. 2006; Wessel and Aron 2013; Swann et al. 2009; Fonken et al. 2016; Wagner et al. 2018). These areas may be expected to show an increase in alpha- and beta-amplitudes during increased motor inhibition, representing a temporary withholding of movement under the state of high motor readiness.

Theta-band oscillations, in contrast, have been found to increase over mid-frontal electrodes during periods of sustained attention (Angelidis et al. 2018; Rajan et al. 2018; Basar-Eroglu et al. 1992; Klimesch 1999), and have been hypothesised to be a correlate of cognitive effort or fatigue (Arnau et al. 2017). We, therefore, assumed that oscillatory power in the theta band may be involved in the attentional, or top-down processes required during effortful tasks.

The present study combined a modified sustained vigilance task (Massar et al. 2016) with a monetary incentive delay task (Knutson et al. 2000) to examine the effects of varying levels of rewards and the value of effort on cortical activation and inhibition. The vigilance task required participants to execute speeded reaction-time (RT) responses during a stream of visual cues occurring in short iterations. It has been shown that requiring participants to complete a sustained vigilance task, with each block offering different rewards (no reward, low reward, or high reward) for each fast response (faster than the participant’s median RT) results in reward-related changes in task performance and sympathetic arousal (Massar et al. 2016), however the effects of reward on cortical oscillatory activity during this task has not yet been investigated.

Experiment 1 aimed to analyse the change in amplitudes of cortical alpha, beta, and theta oscillations in the time-window just preceding the cue prompting a speeded response during a vigilance task, and to test whether individual subjective values of effort, evaluated using a COGED method, would correlate with performance and cortical oscillatory changes. Stimuli were presented in three blocks, with each differing in the incentive for fast responses (0p, 1p, 10p), and EEG data was recorded over a 90-s time window preceding each block to take the baseline into account during the calculation of relative-band power (RBP). Due to this block design, and as participants did not know when the target stimulus would occur, a constant state of motor activation was required, meaning a greater likelihood of observing a modulation of inhibition in cortical oscillatory changes was expected, as the release of inhibition would be required for movement. We, therefore, hypothesised that reward and response-speed would modulate sensorimotor alpha-band and frontal beta-band synchronisation, with stronger synchronisation being found preceding fast trials and in larger reward blocks, representing stronger inhibition.

Since the type of baseline employed in experiment 1 cannot fully account for fast changes in arousal and motivation occurring during a lengthy vigilance task, experiment 2 was carried out to analyse the effect of reward on cortical activation in a vigilance task using a standard event-related desynchronization (ERD) paradigm (Pfurtscheller and Aranibar 1977). The time course of the relative band power changes was analysed in the seconds preceding each trial. Trials involving no reward (0p), a small reward (1p) and a high reward (10p) were presented in a random order, with a visual cue 2 s before the stimulus prompting a speeded response. In this experiment, we aimed to measure the cortical processes associated with motor activation. As the participants knew when the target stimulus would occur, we predicted fast response-speeds and higher rewards would be associated with stronger alpha- and beta-band ERD over sensorimotor regions, as well as stronger theta-band synchronisation over central frontal regions. We also predicted, in both experiments, that participants who showed less effort-discounting in the COGED task would show stronger changes in RT and ERD/RBP as a function of reward.

## Methods

### Experiment 1

#### Participants

29 subjects (12 females) were recruited. Five subjects were removed from subsequent EEG analysis due to excessive muscle artefacts. Therefore, the final sample included 24 participants (10 females), aged 23.34 ± 2.44 (mean ± SD). The procedure used was approved by the Research Ethics Committee of the University of Liverpool and all participants gave fully informed written consent at the start of the experiment in accordance with the Declaration of Helsinki.

#### Procedure

Participants were required to complete two tasks. The participants first completed a modification of the sustained vigilance tasks used by Massar et al. (2016) and Dinges and Powell (1985), while EEG was recorded. The second task was a short discounting task requiring the participants to make a series of 36 choices between a high-effort, high-reward option and a low-effort, low-reward option. The purpose of this task was to estimate the subjective value (SV) attributed to each level of effort offered during the task and to evaluate individual indifference points equalling monetary value and units of effort.

The vigilance task consisted of one 5-min practice block with no EEG recordings and three 10-min experimental blocks with EEG recordings included. The five-minute block consisted of 50 trials, and each ten-minute block consisted of 100 trials. Overall, the participants completed 350 trials throughout the experiment. Participants were offered different rewards for each fast response in each block (0p, 1p, or 10p), and feedback regarding the amount of money and number of points the participants had currently earned was given after each block. Effort was measured behaviourally using the participants’ mean RTs and electrophysiologically using the participants’ change in RBP in the 1-s epoch preceding the presentation of the target stimulus and during the 90 s baseline period of each block.

### Sustained vigilance task

**Fig. 1:**
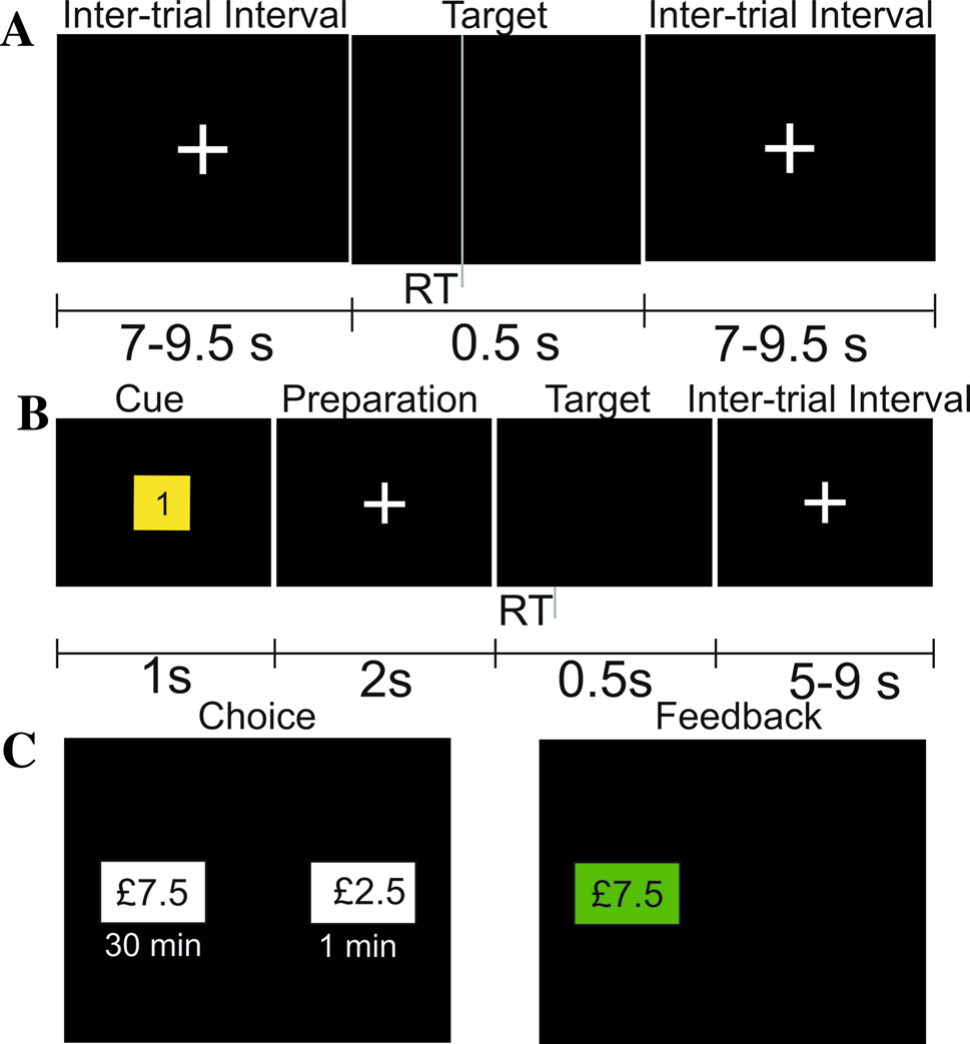
A schematic representation of trials presented to participants in the motivated vigilance task for **a** experiment 1 showing first the inter-trial interval, then the target stimulus, followed by the inter-trial interval for the following trial; **b** experiment 2, showing first the cue stimulus, then the period of preparation, followed by the target stimulus; and, the inter-trial interval, and **c** the discounting choice task for both experiments, showing, first an example choice offered to the participants, followed by feedback confirming the selected choice.

The sustained vigilance task was an adaptation of the Psychomotor Vigilance Test used by Dinges and Powell (1985). This was a 10-min sustained attention task in which participants were required to respond with a button press (left mouse button) with their right hand as quickly as possible whenever they are presented with a target stimulus. The scheme of the vigilance task is shown in Fig. 1a.

After the application of the EEG net, participants were taken into a dimly lit, sound attenuated room and were asked to complete the sustained vigilance task. Participants were seated in front of a 19-inch CRT monitor and used their right hand to make responses on a computer mouse. The stimuli were presented using Cogent 2000 software (UCL, London, United Kingdom) for Matlab R2016b. (Mathworks, Inc., USA).

Participants were presented with a white fixation cross in the centre of a black screen monitor. The target stimulus occurred when the fixation cross disappeared for 0.5 s. The presentation of the target stimuli was separated by uniformly distributed inter-trail intervals which ranged from 3.5 to 9 s. Participants first completed a 5-min practice run of the task with no rewards offered. During this baseline run the participants’ median RT was calculated, which was then used as the target RT in the following 3 10-min blocks.

Following the practice block, participants were required to complete three experimental ten-minute blocks of the same task. In one of the experimental blocks the participants were not offered any reward and were instructed to respond as quickly and as accurately as possible whenever the target stimulus occurred, and in the other two experimental blocks the participants were offered a monetary reward whenever they responded to the target stimulus faster than, or as fast as, their previously calculated median RT. In one of these two blocks participants were offered 1p per fast response and were offered 10p per fast response in the other block. Participants were presented with 100 target stimuli in each block, meaning they were offered a total of £1 or £10 in the two reward blocks, respectively, if they received the reward on every trial. In order to prevent practice or fatigue effects the order of the three experimental blocks was randomly generated by a computer at the start of each experiment, and a one-sample chi-square test was conducted to check the transitional probability of block order, confirming that any block order was not presented significantly more often than the others (*p* = 0.40).

EEG recordings were acquired throughout the study. At the start of each of the three blocks, a 90-s baseline period was recorded, during which participants were instructed to look at the fixation cross presented on the monitor. The cross would not disappear and the participants were not required to make a response.

Trials were split in half based on whether participants responded faster than their median RT were encoded as fast trials and trials where participants responded slower than their median RT were encoded as slow trials. Behavioural measures of attention were taken as being the mean RT for each participant in each experimental block (0p, 1p, 10p) and response-speed trials (fast and slow).

### Discounting task

The discounting task (Massar et al. 2016; Westbrook et al. 2013) was used to evaluate subjective costs of six levels of effort (5, 10, 15, 20, 25, and 30 min) for each participant using a series of monetary decisions.

Participants were first told that they would be required to complete the previous sustained vigilance task again for an amount of time (ranging from one minute to thirty minutes) based on the choices made in the discounting task.

Following this, participants were presented with 36 pairs of monetary offers, with each pair always consisting of one low-effort, low-reward option, and one high-effort, high-reward option (Fig. 1c). The low-effort option always required participants to complete the task again for only one minute, whereas the amounts of time given in the high-effort option was varied based on which condition the trial was in. Participants were offered a fixed reward of £12 in the high-effort option in every trial. In comparison, the reward offered for the low-effort option was adjusted following a staircase titration method (i.e., the offer was increased if the high effort option was chosen and decreased if the low effort option was chosen). The participants were first offered £6 for the low-effort choice with an extra £2.50 being added to, or taken away from, this amount depending on participant choice. The amount of money added to, or taken away from, the low-effort option was then halved each time the participant made a decision. The participants made six choices during each effort block (5, 10, 15, 20, 25, 30 min), and the order of conditions was randomly presented for each participant.

Following the final choice, one trial was randomly chosen through the generation of a random number between 1 and 36, which would then refer to the chosen trial number. Next, the participant would be required to complete the vigilance task for the amount of time chosen during the selected trial and would receive the amount of money associated with that choice.

An indifference point was calculated for each condition, and used as a measure of the subjective value of effort. This was defined as the average of the largest low-effort monetary offer for which the participant chose the low-effort option, and the lowest low-effort monetary offer for which the participants chose the high-effort option (Massar et al. 2016; Westbrook et al. 2013).

In order to control for temporal discounting, participants were informed that they would be required to remain in the laboratory for the full 30 min in total, including the time spent completing the task. This ensured that the participants made decisions during the discounting task based upon the effort required rather than the time taken to complete the task. The boredom associated with remaining in the laboratory was not explored directly; however, all participants discounted higher levels (30 min) more than lower levels (5 min).

The area under the curve (AuC) in the function representing associations between units of efforts and requested payoffs was computed in every participant (Myerson et al. 2001). This measure corresponds to SV of effort and has been found to be correlated with need for cognition scores (Westbrook et al. 2013). A bivariate correlation was conducted to assess the relationship between this function to RTs and RBP values*.*

### EEG recordings

EEG data were recorded continuously using a 129-channel Geodesics EGI System (Electrical Geodesics, Inc., Eugene, Oregon, USA) with a sponge-based HydroCel Sensor Net. The net was aligned with reference to three anatomical head landmarks: two preauricular points and the nasion landmark. Electrode-to-skin impedances were kept below 50 kΩ and were kept at equal levels across all electrodes. A recording band-pass filter was set at 0.001–200 Hz with a sampling rate of 1000 Hz. The Cz electrode was used as a reference electrode.

### Spectral analysis of EEG signals

EEG data were pre-processed using BESA v 6.1 (MEGIS GmbH, Germany). EEG signals were re-referenced using a common average reference method (Lehmann 1984) which restored the signal at electrode Cz. Eye blinks and electrocardiographic artefacts were removed using principal component analysis (Berg and Scherg 1994). Further, data were visually inspected for the presence of any movement or muscle artefacts, and epochs contaminated with artefacts were excluded from subsequent analysis.

While participants completed all trials behaviourally, the average number of trials accepted for EEG analysis in each condition was: 0p, 53.9 ± 14.0 (mean ± SD); 1p, 54 ± 15.5 (mean ± SD); 10p, 55.8 ± 14.3 (mean ± SD). The average number of accepted trials did not differ across conditions (*p* > 0.05). A recording band-pass filter was set at 0.001–1000 Hz with a sampling rate of 1000 Hz.

Continuous EEG data were split into two sets of 1-s epochs. One set of epochs comprised epochs preceding the disappearance of the fixation cross (− 1.0 to 0.0 s). This set of epochs was used to evaluate the cortical activation preceding the speeded RT response. The other set of 1-s epochs was selected from the 90-s resting period which was recorded at the start of each block. All artefact-free 1-s non-overlapping epochs were used. This set of epochs was used to evaluate the baseline amplitudes of cortical oscillations and was used further to evaluate RBP changes.

EEG signals were down-sampled to 256 Hz. In both epochs, the power spectra were computed in Matlab (The Mathworks, Inc., USA) using Welch’s power spectral estimate method. All epochs comprising one set of epochs were aligned to form a quasi-continuous EEG signals. The power spectral densities were computed from non-overlapping 1-s segments (256 points). Each data segment was smoothed using a Hanning window. The power spectral densities were estimated in the range 1–80 Hz with a frequency resolution of 1 Hz.

The RBP in the alpha (8–12 Hz), beta (16–24 Hz) and theta (4–7 Hz) bands were evaluated in each of three conditions using the classical ERD transformation (Pfurtscheller and Aranibar 1979):

$$D=\left(100*\frac{R-A}{R}\right),$$

where* D* represents the RBP during epochs preceding the disappearance of the fixation cross (*A*) relative to the rest condition (*R*). Positive values of *D* correspond to the relative band power decreases which are considered to signify the presence of cortical activation. In contrast, negative *D* values refer to the amplitude increases of band power or cortical synchronisation.

### Statistical analysis

The differences in the median RT across three blocks and two speed conditions of the vigilance task were compared using a 2 × 3 repeated measures ANOVA with three levels of reward (0p, 1p and 10p) and two levels of response-speed (fast and slow). As participants were rewarded based on whether they beat their median RTs, the two levels of response speed were an integral part of the experimental procedure. These were included in this analysis to confirm the separation of the two trial types and to allow for the investigation of interaction effects between response speeds and reward. For the choice task, the AuC in the function representing associations between units of efforts and requested payoffs was computed in every participant (Myerson et al. 2001). This measure corresponds to SV of effort and has been found to be correlated with need for cognition scores in a previous study (Westbrook et al. 2013).

The RBP changes were investigated separately in alpha (8–12 Hz), beta (16–24 Hz) and theta (4–7 Hz) frequency bands across all 129 electrodes using 2 × 3 repeated measures ANOVAs.

A two-step procedure was used to identify electrodes suitable for further analysis. To remove electrodes with spurious results showing only minimal changes in power from the baseline (e.g., < 1% changes) in each frequency band, T-tests with significance thresholds of 0.01 were used to test whether RBP changes over each electrode were significantly different from 0.

Electrode clusters showing statistically significant effects in both the permutation analysis and the *t* tests were explored further in SPSS v. 22 (IBM Inc., USA). The Greenhouse–Geisser epsilon correction was used to tackle a violation of the sphericity assumption found in the data. The correlations between individual RTs and individual changes in RBP were calculated to test for possible covariations between behavioural and electrophysiological effects in all significant electrode clusters.

Further, to tackle the risk of a false positive error due to the large number of tests, a hypothesis-independent permutation analysis, implemented in the *statcond.m* program in the EEGLab package (Makeig et al. 2004), was used to identify clusters of electrodes with significant main effects of reward or response-speed, or interactions between these conditions separately (Maris and Oostenveld, 2007). This cluster-based method provides a data-driven approach to assess effects of conditions on RBP in specified frequency bands (8–12 Hz, 16–24 Hz, and 4–7 Hz) across all electrodes without making a priori assumptions, while also controlling for multiple comparisons with no loss in statistical power.

In this analysis, we calculated the test statistics for the main effects and interactions of both response-speed and reward on RBP in the specified frequency bands over all electrodes. The RBP from all experimental conditions was then collected into a single dataset. Data points were randomly drawn from this set and placed into subsets having the same size as the two response-speed and three reward conditions, forming a ‘random partition’, or dataset representing randomly shuffled versions of the three reward and two response-speed conditions. The test statistics for the main effects and interactions of reward and response-speed in this random partition were then calculated. Next, the creation and analysis of the random partition was repeated 5000 times, and a histogram of the produced test-statistics was constructed for all electrodes. The proportion of random partitions that resulted in a larger statistic than the test-statistic first calculated for the non-shuffled data was calculated for all electrodes, and this was defined as the p-value. Electrodes that exceeded a predefined threshold on the calculated p-values (uncorrected *p* < 0.01) for the main effects of, or interactions between, reward and response-speed were selected and clustered based on spatial adjacency.

## Experiment 2

### Participants

17 subjects (12 females), aged 24.05 ± 3.65 (mean ± SD) were recruited. The procedure used was approved by the Research Ethics Committee of the University of Liverpool, and all participants gave fully informed written consent at the start of the experiment in accordance with the Declaration of Helsinki.

### Procedure

The procedures employed in experiment 2 were identical to those used in experiment 1 except for the structure of the blocks and the trials. The participants first completed an EEG experiment; completing a sustained vigilance task, which was a modification of the vigilance task used in experiment 1 (Dinges and Powell 1985; Massar et al. 2016). Participants then completed the same discounting task as the one employed in experiment 1.

Participants were first presented with a white fixation cross (baseline period) followed by a cue stimulus which displayed the reward value of the next target stimulus (0p, 1p, or 10p) the fixation cross was then displayed in the centre of the screen. After 2.5 s the target stimulus occurred (the fixation cross would disappear for 0.5 s). The presentation of the baseline period and the cue stimulus was separated by uniformly distributed inter-trial intervals which ranged from 3.5 to 9 s and the cue stimulus was presented for 1 s (Fig. 1b). The participants first completed a practice block of the test which lasted for 15 trials with no rewards offered. The participants’ median RT was calculated during the practice block and was then recalculated separately for each reward condition following each trial in the experimental portion of the task.

Following this baseline block, participants were presented with target stimuli in groups of three, containing one trial from each reward condition (0p, 1p, and 10p). The order of trials was pseudo-randomly rearranged at the start of each set of three trails, meaning that the participants could not predict the order of presentation of trials and that there were an equal number of trials in each reward condition presented throughout the duration of the experiment. In the 0p condition participants were offered one point rather than a monetary reward whenever they responded to the target stimulus faster than (or as fast as) their previously calculated median RT. In two of the reward conditions participants were offered a monetary reward whenever they responded to the target stimulus faster than (or as fast as) their previously calculated median RT. Participants were offered 1p per fast response in one condition, and were offered 10p per fast response in the other. The participants were presented with 100 target stimuli for each condition, meaning that the participants were offered a total of £0, £1 or £10 across all the trials in each reward condition. During the baseline periods of the experiment, participants were instructed to look at the fixation cross presented on the monitor without making a response.

Trials were divided in half, whereby trials which participants responded faster than their median RTs were encoded as fast trials and trials where participants responded slower than their median RTs were encoded as slow trials. Behavioural measures of attention were taken as being the mean RTs for the participants in each experimental block (0p, 1p, 10p) and response speed condition (fast, slow). The average number of trials in each condition was: 0p 73.67 ± 14.62 (mean ± SD); 1p 76.76 ± 12.84 (mean ± SD); 10p 74.95 ± 11.53 (mean ± SD). The average number of trials accepted did not differ across conditions (p > 0.05). Fewer trials were removed from the EEG analysis in this experiment compared to experiment 1 due to overall cleaner data.

### Event-related desynchronization analysis

ERD in alpha, beta and theta bands was computed at every electrode by first calculating the absolute band-power value from 1-s time epochs shifted in 100-ms steps across a 9-s trial window. The trial time window ranged from 2 s before and 7 s after the onset of the cue signalling the amount of reward. The power spectral densities in every one of the 81 time-bins were computed using the Welch method. Each data epoch was smoothed using a Hanning window. The epoch ranging from − 1.5 to − 0.5 s was used to evaluate rest amplitudes of cortical oscillations and this value was used to compute ERD at every time point across the trial according to the ERD transform (Eq. 1). ERD values in the time epoch ranging from 2 to 3 s after the cue onset and immediately preceding the disappearance of the fixation cross were averaged for further statistical analysis.

### Statistical analysis

The differences in the median RTs across three blocks and two speed conditions of the vigilance task were compared using a 2 × 3 repeated measures ANOVA with three levels of reward (0p, 1p and 10p) and two levels of response-speed (fast and slow). For the choice task, each participant’s indifference point was calculated for each effort block (5, 10, 15, 20, 25, 30 min).

ERD was investigated in theta (4–7 Hz), alpha (8–12 Hz) and beta (16–24 Hz) frequency bands across all 129 electrodes using 2 × 3 repeated measures ANOVA. To tackle the risk of a false positive error due to the large number of tests *p* values were corrected using a permutation analysis (Maris and Oostenveld 2007), implemented in the *statcond.m* program in the EEGLab package (Makeig et al. 2004). To prevent multiple comparisons from creating false effects, electrode clusters were selected using a permutation analysis with 5000 permutations. Electrodes with statistically significant main effects or interactions were selected for further analysis. *T* tests with significance thresholds of 0.001 were used to test whether ERD over each electrode was significantly different from 0. Only electrodes which passed significance thresholds in both tests were selected for subsequent analysis. The combined statistical and amplitude threshold ensured that results were extracted only from electrodes showing task-related responses.

Electrode clusters showing a statistically significant effects in both the permutation and *t* test analyses were explored further in SPSS v. 22 (IBM Inc., USA). Greenhouse–Geisser epsilon correction was used to tackle the violation of the sphericity assumption due to more than two levels in the independent variable.

To test possible covariations between band power, RT changes, and individual SVs, difference variables were created. These were defined as the mean difference between fast and slow trials for each participant, which were calculated by subtracting fast trial RTs and RBP from slow trial RTs and RBP power. The RBP and RT difference variables were correlated with each other and individual AuC of SVs using bivariate correlations. Bivariate correlations were conducted in all electrode clusters or single electrodes selected for further analysis; however, only statistically significant correlation coefficients are reported.

## Results

### Experiment 1

#### Vigilance task

**Fig. 2:**
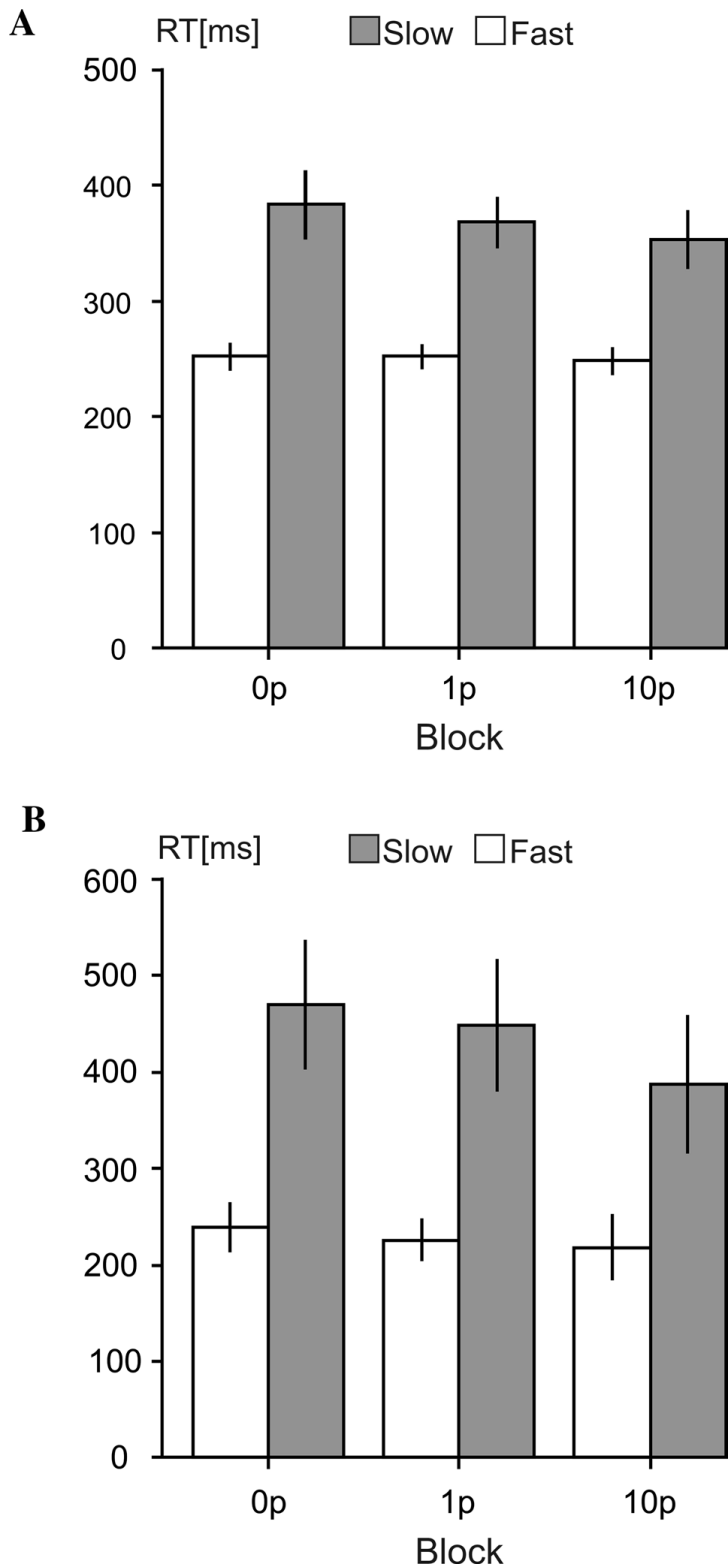
A bar chart to show the mean RTs in each reward condition (0p, 1p, 10p) in slow (grey) and fast (white) trials in experiment 1 (**a**) and experiment 2 (**b**). Error bars represent the standard errors of the mean

Differences in median RTs across the three reward conditions (0p, 1p, 10p), and across fast and slow trials were analysed using a 2 × 3 repeated measures ANOVA. A statistically significant main effect of reward was found (*F*(2,56) = 6.75, *p* = 0.003, ${\eta p}^{2}$ = 0.19) with a significant negative linear trend (*p* = 0.001). This was found to be the result of a difference between the 10p reward block and both the 1p (*p* = 0.047) and the 0p reward blocks (*p* = 0.001). Median RTs in slow and fast trials in each reward category are shown in Fig. 2a.

A statistically significant interaction between reward and response-speed was also found (*F*(2,56) = 5.03, *p* = 0.012, ${\eta p}^{2}$ = 0.15). A test of simple effects showed that this interaction was due to an effect of reward on RTs for slow trials only (*F*(2,46) = 7.15, *p* = 0.003) with a statistically significant negative linear trend (*p* = 0.002). The main effect was found to be the result of a difference between the 10p reward block and both the 0p (*p* = 0.001) reward block. No statistically significant effect of reward was found for fast responses.

**Fig. 3:**
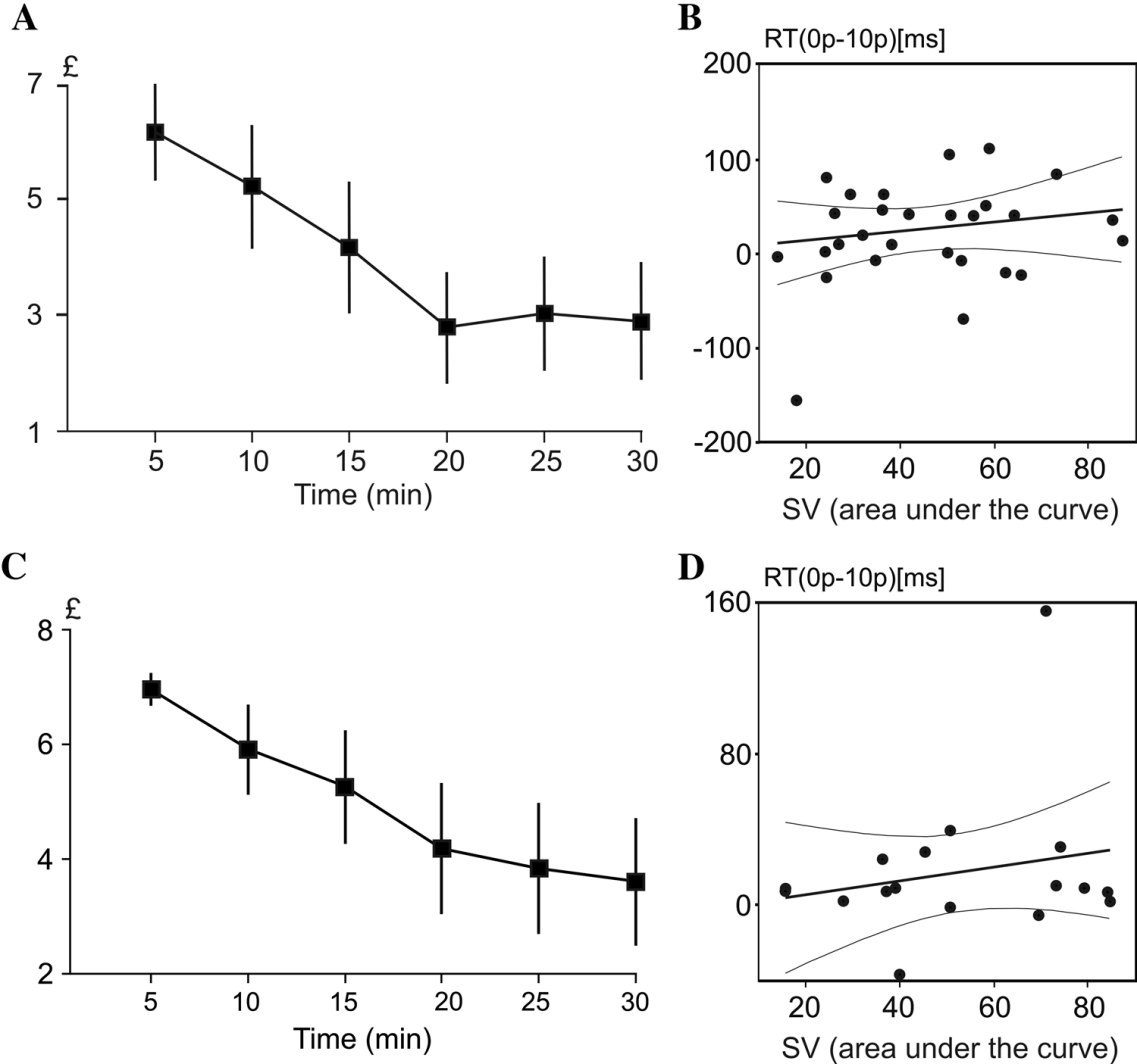
A line graph to show the discounting curve in the choice task, with the mean subjective value shown for each block in the task (5, 10, 15, 20, 25, 30 min). A discounting curve is shown for both **a** experiment 1 and for experiment 2 (**c**). Error bars represent standard errors of the mean. Scatterplots to show the correlation between the area under the curve of SVs in the discounting task and the median RTs difference between high-reward and no reward conditions (0p-10p) for experiment 1 (**b**) and experiment 2 (**d**)

RT difference variables were correlated with the value of effort evaluated as AuC in individual COGED graphs representing amount of money to be paid for each of the six task durations, with no statistically significant correlation being found between RT changes and individual SVs of effort (see Fig. 3b).

### Discounting task

A linear regression analysis was used to compare the change in SV for each effort condition (5, 10, 15, 20, 25 and 30 min). The mean discounting values across offered 5–30 min task durations are shown in Fig. 3a. There was a statistically significant exponential relationship between the levels of effort and SVs (*F*(1, 172) = 32.87, *p* < 0.001, ${R}^{2}$ = 0.17). The regression model showed a negative exponential regression with an equation of:

$$Y \, = \, 6 \, \times \, \exp \, \left( { - 0.041 \, \times \, X} \right) \, + \, \varepsilon ,$$

where Y is the SV, X is the effort level, and ε is an error element.

### Alpha-band changes

**Fig. 4:**
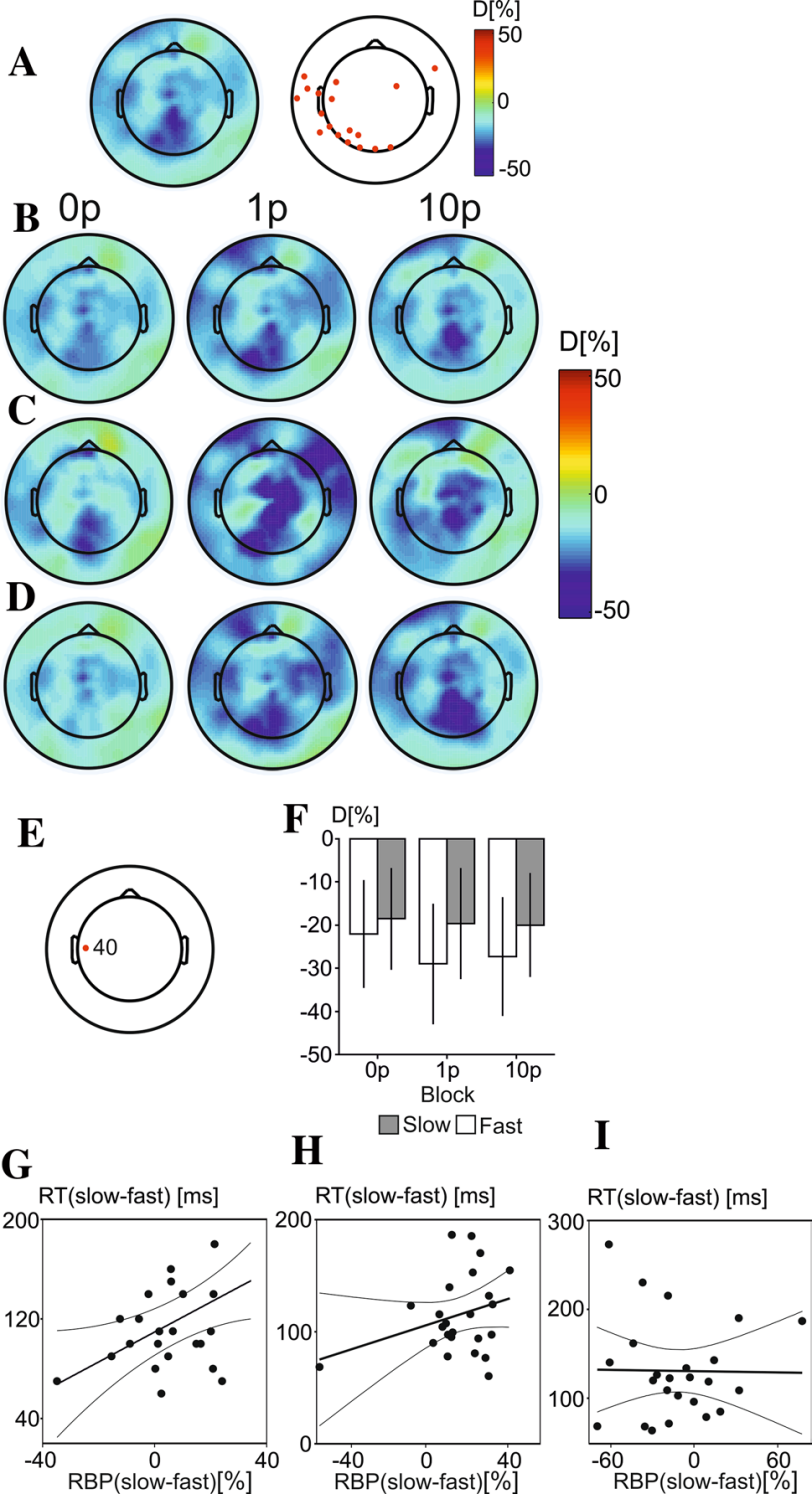
The RBP changes in alpha band in experiment 1. **a** A grand average topographic map of alpha-RBP averaged across all conditions and subjects. **b** An overhead view of electrodes showing statistically significant changes in alpha band across all conditions. **c** Grand average topographic maps of alpha-RBP in 0p, 1p and 10p conditions during trials with slow RTs. **d** Grand average topographic maps of RBP in three reward conditions in fast RT trials. **e** Grand average topographic maps of alpha RBP in three reward conditions across all trials and the location of electrode 40 showing an interaction between reward values and speed of motor response. **f** The mean values of alpha RBP in slow (grey rectangles) and fast (white rectangles) in three reward conditions at electrode 40. The error bars represent standard errors of the mean. Scatter plot and linear regression lines representing correlation between the difference alpha RBP (slow–fast trials) and the difference RT (slow–fast trials) at electrode 40 in 10p condition (**g**), the 1p condition (**h**), and the 0p condition (**i**)

Figure 4a shows the grand average topographic maps of RBP over all trials (left), as well as the electrodes found to be different from 0 (right). Electrodes responding with amplitude changes in the alpha band included the posterior parietal and occipital cluster of electrodes, the left central-temporal cluster, and two electrodes over the right frontal and prefrontal region of the scalp. The grand average topographic maps of RBP in each of the three reward conditions are shown for slow (Fig. 4b) and fast (Fig. 4c) trials, as well as across all trials (Fig. 4d).

The topographic maps show widespread increases in alpha RBP, with larger RBP increases preceding fast compared to slow trials over left-central region of the scalp. Electrode 40, over the left-central area, was the only electrode found to pass both the difference from 0 *t* test and the permutation-based threshold, and was, therefore the only electrode selected for further analysis. To investigate RBP changes over this electrode a 2 × 3 repeated measures ANOVA was conducted, with 3 levels of reward (0p, 1p and 10p) and 2 levels of response-speed (fast and slow). A significant main effect of response-speed was found (*F*(1,23) = 4.37, *p* = 0.048), where fast responses were found to elicit significantly stronger synchronisation compared to slow responses. Electrode location is shown in Fig. 4e and RBP values for electrode 40 are shown in Fig. 4f.

In order to assess the relationship between RBP changes and RTs, difference variables were created. These were defined as the mean difference between fast and slow trials for each participant, being calculated by subtracting fast trial RTs and RBP from slow trial RTs and RBP power. There was a significant positive correlation between alpha RBP and RT difference variables in the 10p reward block (r(24) = 0.42, *p* = 0.015), showing that participants with stronger synchronisation in fast relative to slow trials had shorter RTs in fast relative to slow trials. However, no significant correlations were found between the same RT and RBP difference variables created in either the 0p (*r*(24 = − 0.015, *p* = 0.95), or 1p (*r*(24 = 0.29, *p* = 0.15)) reward blocks. Results of these correlations are shown in Fig. 4g–i.

The changes in alpha RBP were also correlated with the value of effort evaluated as AuC in individual COGED graphs representing amount of money to be paid for each of the six task durations. However, no statistically significant correlation was found between alpha-band power changes and individual SVs of effort acquired in COGED task.

### Beta-band changes

**Fig. 5:**
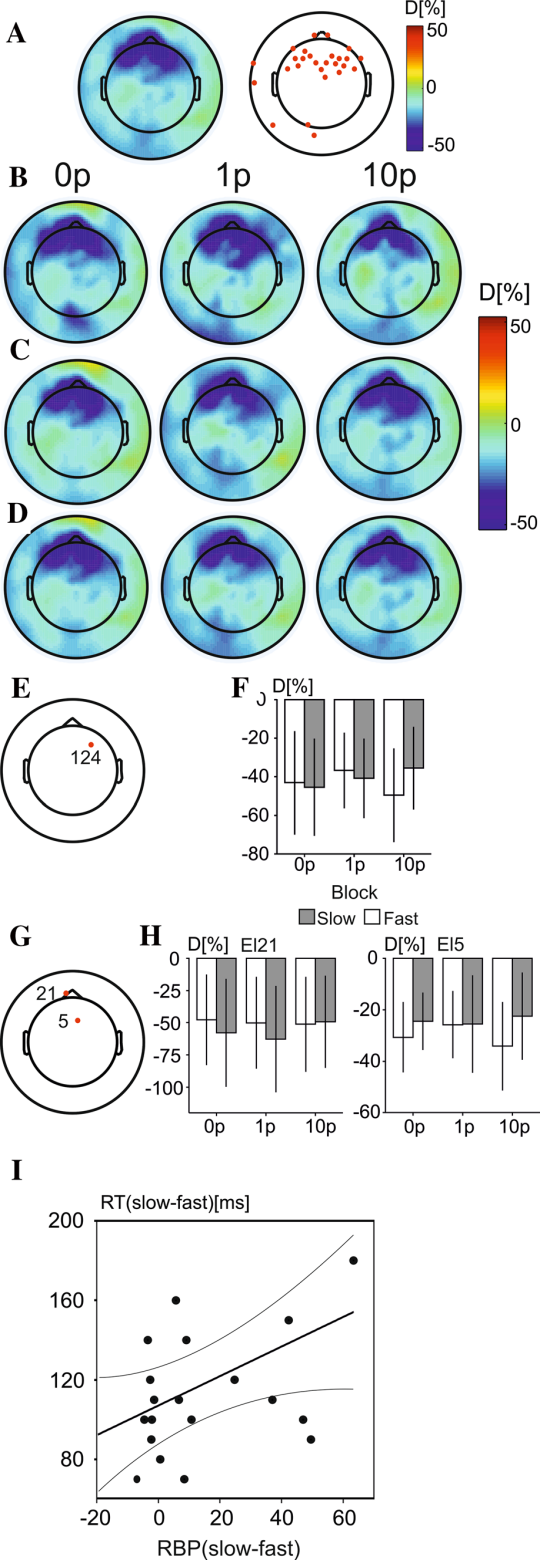
The relative band power changes in beta band in Experiment 1. **a** Grand average topographic map of beta RBP across all conditions and subjects. **b** An overhead view of electrodes showing statistically significant changes in beta band across all conditions. **c** Grand average topographic maps of beta RBP in 0p, 1p and 10p conditions during trials with slow RT. **d** Grand average topographic maps of beta RBP in three reward conditions in fast RT trials. **e** Grand average topographic maps of beta RBP in three reward conditions across both slow and fast RT trials. E. Location of electrode 124 showing an interaction between reward values and speed of motor response. **f** The mean values of beta RBP in slow (grey rectangles) and fast (white rectangles) in three reward conditions at electrode 124. The error bars stand for standard errors of the mean. **g** Locations of electrodes 121 and 5 showing a statistically significant main effect of response speed. **h** The left-hand panel shows mean beta RBP at electrodes 121 and 5 in three reward conditions for slow (grey rectangles) and fast (white rectangles) trials. **i** The scatter plot and linear regression line with 95% confidence interval lines depicting association between differences in RT (slow–fast trials) and differences beta-band RBP (slow–fast trials)

Figure 5a (right panel) shows the grand average topographic maps of beta RBP over all trials (left), showing strong increases in RBP over frontal regions of the scalp at electrodes surpassing a combined statistical and amplitude threshold highlighted with red circles (left panel). The grand average topographic maps of relative band power in each of the three reward conditions are shown for slow (Fig. 5b) and fast (Fig. 5c) trials as well as across all trials (Fig. 5d). Three electrodes passed both the difference from 0 and the permutation-based threshold and were, therefore, selected for further analysis.

A statistically significant interaction between reward and response-speed was found over the right-frontal region of the scalp (electrode 124) (*F*(2,46) = 4.51, *p* = 0.016). The interaction was found to be due to an effect of response-speed in the 10p reward block (*F*(1,23) = 9.37, *p* = 0.006), where fast responses were found to elicit statistically significantly more beta-band synchronisation compared to slow responses. Electrode location is shown in Fig. 5e and mean values of beta-band RBP in all conditions are shown in Fig. 5f.

A statistically significant main effect of response-speed was found over a frontal electrode (electrode 21) (*F*(1,23) = 5.64, *p* = 0.026), where fast responses were found to elicit significantly weaker beta band synchronisation compared to slow-responses. In contrast, electrode 5, located in the midline fronto-central area of the scalp (Fig. 5g), showed a stronger beta-band power increase in fast compared to slow responses (*F*(1,23) = 9.23, *p* = 0.006) (Fig. 5h).

To evaluate the relationship between RTs and RBP over right-frontal regions (electrode 124) a difference variable was calculated in both RTs and RBP values representing the differences between fast and slow trials in the 10p reward block only, being calculated by subtracting fast trial RBP and RTs from slow trial RBP and RTs. The Pearson product-moment correlation showed a statistically significant positive relationship between the difference values computed for RTs and RBP over electrode 124 (*r*(24) = 0.44, *p* = 0.033) (Fig. 5i). This shows that participants with a stronger increase in beta-band power in fast trials compared to slow trials in the 10p reward bock also had a greater difference in RTs between slow and fast trials in this block. No significant correlation was found between RBP changes in the beta band and individual discounting results.

Data were also analysed in the theta frequency band; however, no electrodes were found to pass both significance thresholds in this frequency range.

### Absolute band power changes

In order to confirm that the effects found within the alpha- and beta-bands were not the results of changes in baseline power, the absolute power of the baseline conditions was compared over relevant electrodes in the alpha- and beta-bands. No significant differences in baseline were found across reward conditions for any of the relevant electrodes (*p* > 0.05) in either frequency band, confirming that the results of experiment 1 were not the result of variations within the baseline power.

## Discussion

The results of experiment 1 show that the presence of monetary incentives shortened RTs, and fast responses were associated with stronger synchronisation in the alpha band over the left-central area of the scalp and stronger and more focused synchronisation in the beta band over fronto-central regions of the scalp, an effect which was particularly apparent in high-reward conditions. Individual values of subjective effort, however, were not associated with band-power increases in either the alpha or beta frequency bands. Thus, we were unable to replicate the correlation of *r* = 0.31 between the value of effort and the shortening of RTs found in previous research (Massar et al. 2016). However, the order of the three reward blocks was randomised in the present study, whereas in previous research the no reward block was always presented first. This procedural difference may explain the lack of a statistically significant correlation between the individual value of effort and performance.

The effects of response-speed were seen as modulations of amplitude increases in both alpha- and beta-band power in the 1-s epoch preceding the motor response, compared to the baseline. In the alpha band, a stronger increase in oscillatory power was observed in fast compared to slow trials over a left-central electrode. This effect was significantly correlated with the individual differences between fast and slow mean RTs in the 10p reward block. An effect of reward was present only in the beta band, as a stronger synchronisation of beta-band oscillations prior to fast compared to slow responses in 10p condition but not in 0p or 1p conditions. Individuals with the largest differences between slow and fast RTs also showed the strongest increase in beta-band power at the frontal electrode.

Amplitude increases in the alpha-band over central regions have traditionally been associated with motor inhibition (Fry et al. 2016; Jensen et al. 2005; Pfurtscheller et al. 1996; Salmelin and Hari 1994). This is thought to be due to the absence of excitatory impulses from lower brain centres (e.g., the reticular formation) (Zaaimi et al. 2018; Steriade and Demetrescu 1962; Bonvallet and Newman-Taylor 1967) and due to the synchronised firing of GABAergic neurons (Faust et al. 2016; Tritsch et al. 2016; Jensen et al. 2005; Klimesch et al. 2007).

Beta-band increases were stronger and more focused over fronto-central regions preceding fast responses compared to slow responses, reflected in a different pattern of ERD changes in electrodes 5 and 21. A similar pattern of a prominent fronto-central focus of beta-band synchronization due to topographic expansion has been found for Go, compared to NoGo, responses (Alegre et al. 2004). While our data do not allow inferences on underlying cortical generators, the shape differences in the large ERD cluster in prefrontal and fronto-central electrodes suggests that the fast- compared to slow movements were preceded by a stronger activation in premotor regions residing in the medial frontal cortex. This interpretation is supported by findings of activations in the right frontal cortex during stop-signal and Go/No Go task, and of increased beta-band synchronisation over frontal electrodes during motor inhibition (Alegre et al. 2006; Wessel and Aron 2013; Swann et al. 2009; Fonken et al. 2016; Wagner et al. 2018). The pattern of cortical oscillations in experiment 1 matched the inhibitory processes posited by the horse-race theory (Logan and Cowan 1984; Logan 1994; Band et al. 2003), showing that active inhibition was required during motor preparation and that this was modulated by response-speed, especially under conditions of high reward.

Both the alpha- and beta-band results suggest faster response speeds, especially under high reward, were associated with increased motor inhibition in the time window preceding movement. This relates to the experimental design, where the target was not cued, so motor activation was required to be maintained throughout each block. The increased inhibition found may relate to higher engagement with the task or be due to a faster motor response, and the correlation found between RTs and RBP in the 10p reward block supports this explanation.

## Experiment 2

### Vigilance task

Differences in median RTs in response to the target stimulus were assessed across the 3 reward conditions (0p, 1p and 10p) in both fast and slow trials using a 2 × 3 repeated measures ANOVA. A significant main effect of reward was found (*F*(2,32) = 12.58, *p* = 0.001, ${\eta p}^{2}$ = 0.44), with a significant negative linear trend (*p* = 0.002). This main effect was found to be the result of significant differences between the 10p reward condition and both the 1p (*p* = 0.003) and the 0p (*p* = 0.002) reward conditions. The mean values of RTs in each reward and response-speed conditions are shown in Fig. 2b.

A significant interaction was also found between reward and response-speed (*F*(1,32) = 10.80, *p* = 0.002, ${\eta p}^{2}$ = 0.40) and, in order to investigate this interaction one-way repeated measures ANOVAs assessed the effect of reward on RTs during fast and slow trials separately. The interaction was related to the statistically significant modulation of RTs during slow trials only (*F*(2,32) = 12.84, *p* = 0.001, ${\eta p}^{2}$ = 0.45) with a significant negative linear trend (*p* = 0.001). Further analysis of post-hoc effects revealed a significant difference between the 10p reward condition and both the 1p (*p* = 0.001) and 0p (*p* = 0.001) reward conditions. No statistically significant simple effect of reward on RTs were found in fast trials.

A difference variable representing the high reward RTs subtracted from low reward RTs (10p–0p) correlated with the AuC in individual COGED graphs. However, no statistically significant correlation was found between RT changes and individual SVs of effort acquired in COGED task (see Fig. 3d).

### Discounting task

A linear regression analysis was conducted to compare the change in SV for each block during the discounting task (5, 10, 15, 20, 25, 30 min). There was a significant exponential relationship between the levels of effort and SVs (*F*(5, 15) = 6.66, *p* < 0.002, ${R}^{2}$ = 0.69) (Fig. 3c). The regression model showed a negative exponential regression with an equation of:

$$Y = 6 \times \exp \left( { - 0.041 \times X} \right) + \varepsilon,$$

where* Y* is the SV,* X* is the effort level, and* ε* is an error element.

### ERD patterns across trials

**Fig. 6:**
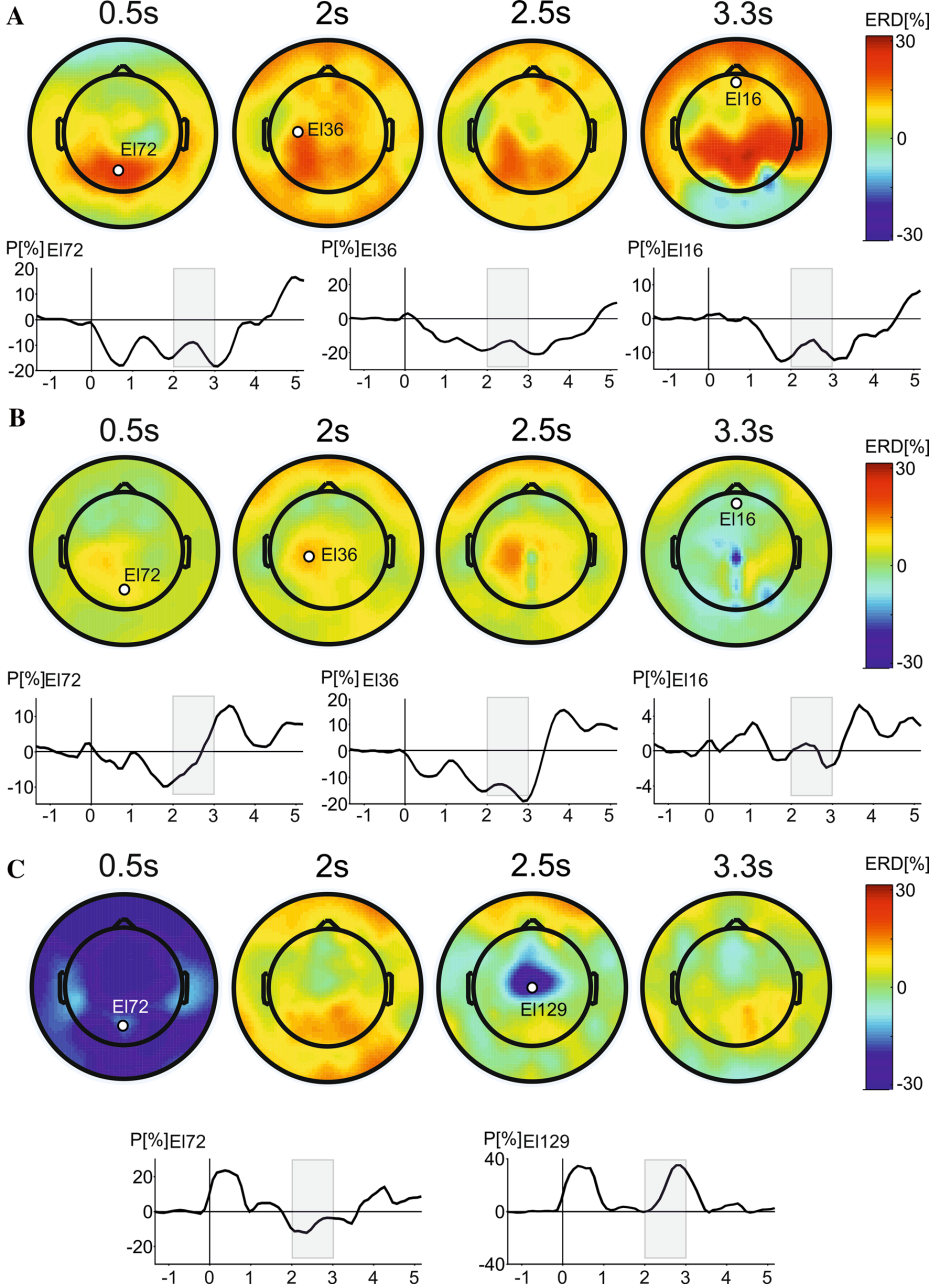
Topographic maps of alpha (**a**), beta (**b**) and theta (**c**) ERD at four time points: during presentation of visual cue (0.5 s), early period of anticipation of motor response (2 s), late period of motor response anticipation (2.5 s) and during motor response (3.3 s). In each section (**a**–**c**), ERDs at selected electrodes are also shown. The grey rectangles covering the interval from 2 to 3 s represent the epoch of interest preceding the motor response

Figure 6 shows ERD/ERS scalp topographies over specified time periods (0.5 s, 2 s, 2.5 s, and 3.3 s following the presentation of the cue stimulus) in (A) the alpha-band, (B) the beta band, and (C) the theta band. Time courses of percentage power changes over specified electrodes are also shown. Electrodes were selected apriori at areas of the scalp where band power was expected to be modulated by task demands based on previous research. For example, an ERD was expected over contralateral sensorimotor areas in the alpha- and beta-bands during motor preparation (Pfurtscheller and Berghold 1989; Tzagarakis et al. 2010, 2015; Fox et al. 2016; Ishii et al. 2019). Oscillations during the cue interval (0.5 s after cue onset) were featured by an ERD over occipital electrodes in the alpha band (Fig. 6a). This is consistent with the presence of attentional and visual processing of a reward cue. During the period of motor readiness (2–2.5 s after cue onset), alpha-ERD was prominent in left (contralateral) parietal, and central electrodes. After the cue disappeared and during the time of motor response, alpha-ERD was distributed bilaterally in parietal, and central electrodes.

In the beta band (Fig. 6b), a comparatively weak ERD appeared in the contralateral central electrodes during the period of motor readiness preceding the disappearance of the fixation cross. A beta-ERS was seen at the vertex electrode during motor preparation (2.5 s after cue onset). This increased during the motor response period (3.3 s after cue onset).

Finally, in the theta band (Fig. 6c), activation during the cue interval (0.5 s after cue onset) was confounded by the presence of the phase-locked evoked response causing an increase of theta power over the whole scalp. The period of motor readiness (2.5 s after cue onset) was featured with a theta-ERS at central and precentral midline electrodes.

### Alpha-band ERD

**Fig. 7:**
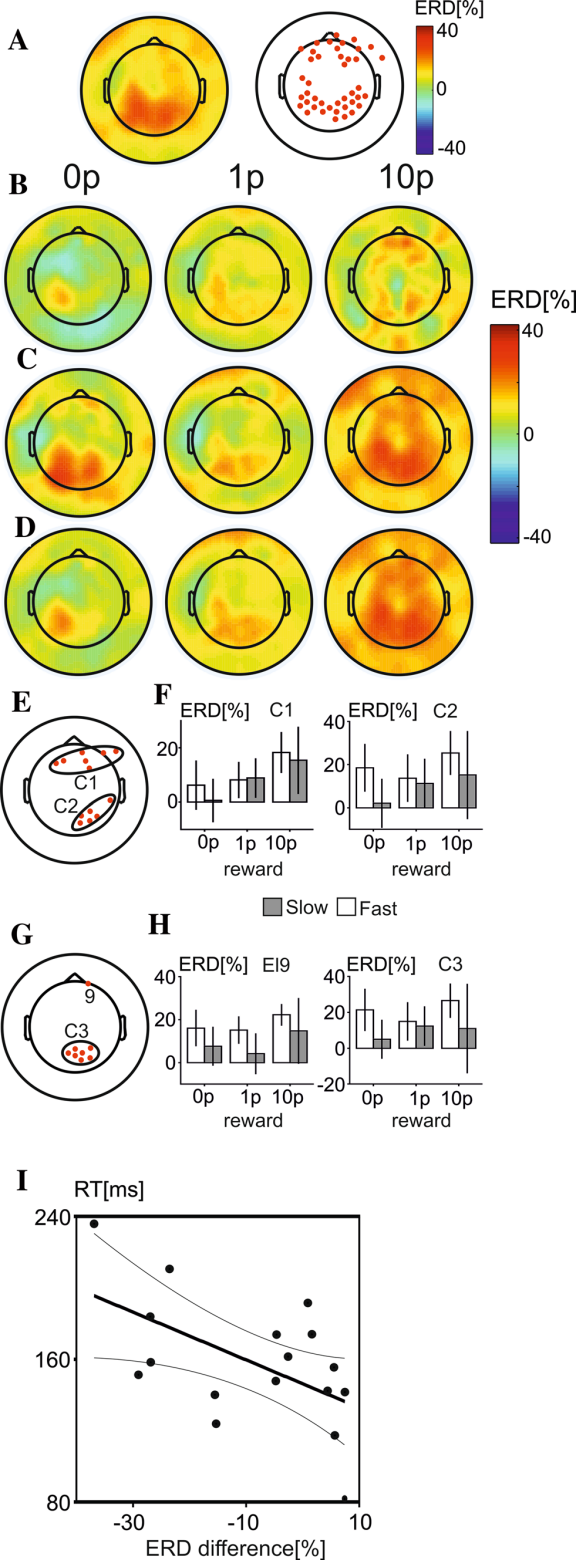
Alpha-band ERD during anticipation of motor response **a** Topographic map of alpha-band ERD across all conditions and trials (left), and electrodes showing a prominent alpha-band ERD across all conditions (right). **b** Topographic maps of alpha-band ERD in three reward conditions during slow ER trials. **c** Topographic maps in each of three reward conditions in fast RT trials. **d** E. Location of electrodes in two clusters manifesting statistically significant effect of reward. **f** Bar charts showing mean alpha-band ERD each of three reward conditions in slow (grey rectangles) and fast (white rectangles) RT trials. Error bars represent standard error of the mean. **g** Locations of electrodes displaying a statistically significant main effect of speed of motor response. **i** A scatterplot and the linear regression line with 95% confidence lines illustrating the statistically significant correlation between alpha-band ERD differences (slow-fast RT trials) and RT differences in electrode 9

The grand average topographic maps showing alpha-band ERD for all trials as well as the electrodes found to be significantly different from zero are shown in Fig. 7a. Two clusters of electrodes, one in bilateral parietal and central electrodes and another in frontal electrodes, showed alpha-ERD surpassing both the combined amplitude and statistical thresholds.

Topographic maps showing ERD in each of the three reward conditions are shown in Fig. 7b for slow, and Fig. 7c for fast trials, and in Fig. 7d for all trials irrespective of the speed of the motor response.

To investigate the effects of response-speed and reward on ERD values 2 × 3 repeated measures ANOVAs were computed to assess the significant main effects and interactions of response-speed (fast and slow) and reward (0p, 1p, 10p) on ERD recorded by electrodes which passed the combined statistical and amplitude thresholds. This ensured that only electrodes showing a robust ERD across conditions were analysed.

Statistically significant main effects of reward were found in both frontal and occipital regions of the scalp. Over frontal electrodes (cluster 1) ERD grew significantly stronger as reward increased (*F*(2,32) = 7.95, *p* = 0.003, ${\upeta \mathrm{p}}^{2}$ = 0.44), and a statistically significant positive linear trend was found (*p* = 0.005). The observed main-effect of reward was due to a difference between ERD in 10p reward trials and both 0p (*p* = 0.005) and 1p reward trials (*p* = 0.008). There was also a statistically significant effect of reward on ERD found over right-parietal regions (cluster 2) (*F*(2,32) = 4.31, *p* = 0.022, ${\upeta p}^{2}$ = 0.31), with a statistically significant linear trend (*p* = 0.017). This effect was found to be the result of a difference between ERD calculated for 10p trials and for 0p trials (*p* = 0.017). Electrodes with a main effect of reward are shown in Fig. 7e, and results for both cluster 1 and cluster 2 are shown in Fig. 7f.

Significant main effects of response-speed were also found over frontal and occipital electrodes, where fast trials were found to elicit significantly stronger ERD when compared to slow trials. There was significantly stronger ERD found over electrode 9 (frontal) during fast trials compared to slow trials (*F*(1,16) = 6.21, *p* = 0.024, ${\eta p}^{2}$ = 0.28), and stronger ERD over cluster 3 (occipital) during fast compared to slow trials (*F*(1,16) = 5.21, *p* 0.037, ${\eta p}^{2}$ = 0.25). Electrodes with a significant main effect of response-speed are shown in Fig. 7g and ERD results for electrode 9 and cluster 3 are shown in Fig. 7h.

A difference variable was created to by subtracting fast from slow trials for both individual ERD values over electrode 9 and individual RTs. A significant negative correlation was found between these two difference variables (*r*(17) = − 0.55, *p* = 0.021), showing that stronger differences in ERD between fast and slow trials were associated with larger differences in RTs between these trials (Fig. 7i).

Difference variables were also created to calculate the mean difference between the ERD found during 10p reward trials and both 1p and 0p reward trials in cluster 1, and to calculate the mean difference in the participant’s indifference points taken from the COGED task during 5 min and 30 min effort conditions. There was, however, no statistically significant correlation between the SV of effort, evaluated as AuC of individual COGED functions, and alpha-band ERD.

### Beta-band ERD

**Fig. 8:**
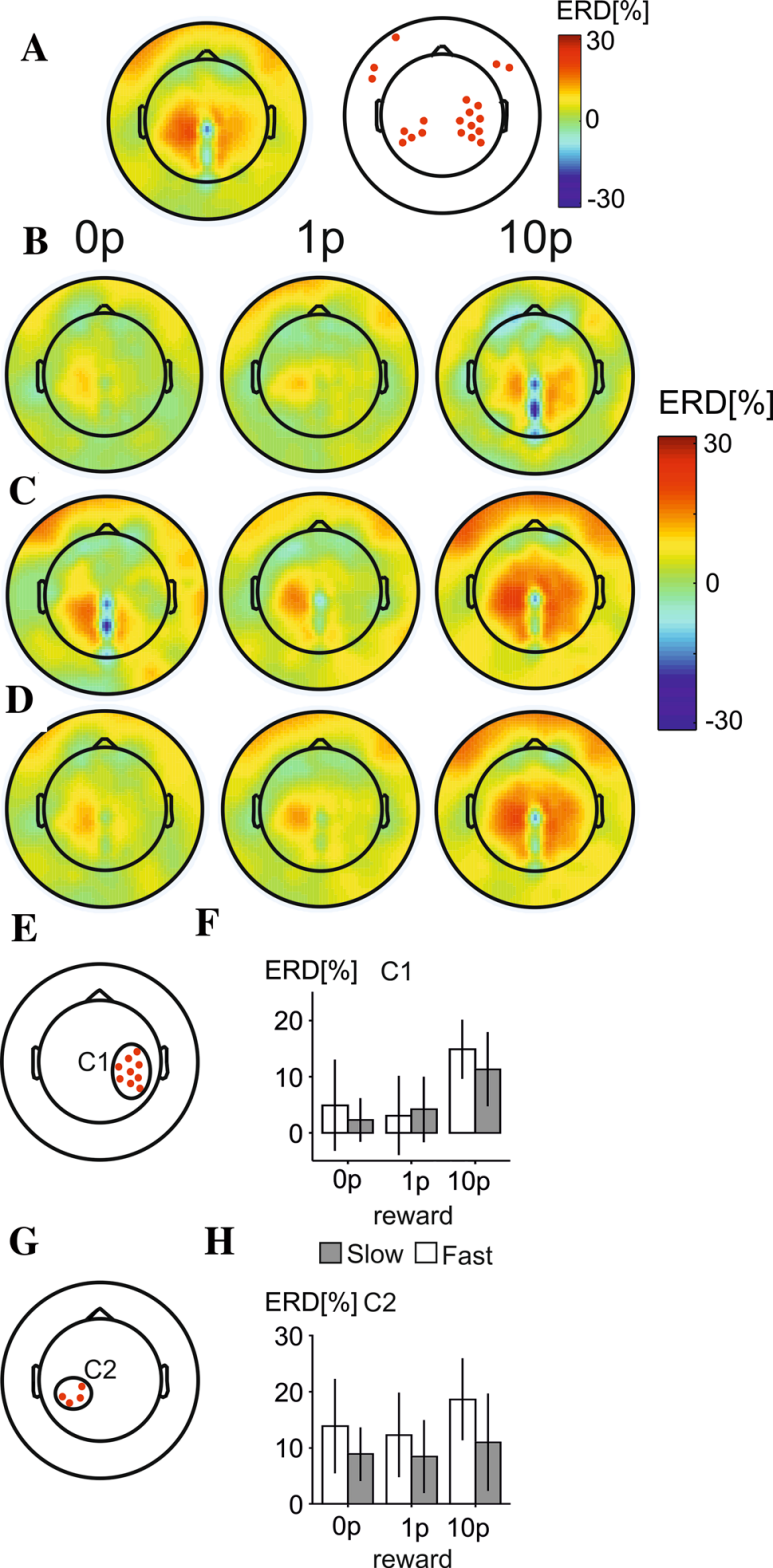
Topographic maps and statistically significant effects in beta-band ERD. **a** Grand average beta-band ERD across all trials and subjects (left panel) and locations of electrode clusters manifesting a statistically significant beta-band ERD (right panel). **b** Topographic maps of beta-band ERD in three reward conditions (0p, 1p and 10p) in slow RT trials. **c** Topographic maps of beta-band ERD in fast RT trials. **d** Topographic maps of beta-band ERD in three reward conditions averaged across fast and slow trials. **e** Location of the electrode cluster, labelled C1, showing a statistically significant effect of reward. **f** Mean values of beta-band ERD in the cluster shown in (**f**) in three reward conditions in slow (grey rectangles) and fast (white rectangles). The error bars stand for standard errors of the mean. **g** The location of electrode cluster, labelled C2, showing a statistically significant effect of speed of motor response. **h** Mean values of beta-band ERD in three reward conditions in slow (grey rectangles) and fast (white rectangles) RT trials

The grand average topographic map for all trials and the distribution of electrodes showing ERD significantly different from zero are shown in Fig. 8a. The electrodes with a strong beta-ERD across conditions were located primarily in the left, right-central and parietal electrodes. The grand average topographic maps in each of the three reward conditions are shown for slow trials in Fig. 8b, for fast trials in Fig. 8c, and for all trials in Fig. 8d.

ERD in the beta band featured a comparatively weak effect in the contralateral central and parietal electrodes in the 0p and 1p conditions compared to the 10p condition. Beta-ERD was also pronounced over ipsilateral central electrodes; however, this effect was only found in the 10p condition. ERS can also be seen over central regions (electrodes Cz–Oz), an effect consistent with the ‘surround ERS’ (Suffczynski et al. 2001) found around areas showing ERD in previous studies (Pfurtscheller 2003; Pfurtscheller et al. 2000; Neuper et al. 2006; Doyle et al. 2005).

There was a significant main effect of reward in the ipsilateral (right) sensorimotor hand area (cluster 1, Fig. 8e) (*F*(2,32) = 10.14, *p* = 0.001, ${\eta p}^{2}$ = 0.58), with a significant positive linear trend (*p* = 0.004) (Fig. 8f). The main effect of reward was related to the statistically significant difference between 10p reward and both the 1p (*p* < 0.001) and 0p reward conditions (*p* < 0.001).

In the contralateral (left) cluster of electrodes (cluster 2, Fig. 8g), beta-band ERD was significantly stronger when preceding fast trials compared to slow trials (*F*(1,16) = 10.39, *p* = 0.005, ${\eta p}^{2}$ = 0.39) (Fig. 8h). There was no effect of reward in cluster 2 (*p* > 0.05).

In order to evaluate the relationship between behavioural results and beta-ERD found ipsilateral to the hand movement a difference variable was created where the mean ERD difference between 10p reward trials and both 1p and 0p reward trials was calculated. However, there was no statistically significant correlation between beta-band ERD and RT difference values. Similarly, there was no statistically significant correlation between beta-band ERD and the SV of effort in any of the electrode clusters (*p* > 0.05).

Similar to experiment 1, there were no statistically significant effects of reward or speed of response in theta band.

## Discussion

Reward level quickened RTs, especially in slow movements. The COGED profiles showed decreasing SVs of reward as the associated effort was increased similar to previous studies (Massar et al. 2016; Westbrook et al. 2013). However, no significant correlation was found between the SV of effort and either RTs or cortical oscillatory changes. We were, again, unable to replicate the correlation between value of effort and RTs found in Massar et al. (2016). It appears that this correlation is difficult to replicate if the order of blocks or trials with different reward levels occurs in a random order, showing independence between the individual value of effort and the way rewards effected the modulation of effort during the vigilance task.

ERD in the alpha band showed reward-related increases, with the strongest ERD in the 10p condition in two clusters of electrodes, one in the frontal and the other the parietal region of the scalp. Both regions also showed a stronger ERD prior to fast, compared to slow motor responses. In the beta-band, ERD was localised in contralateral central regions of the scalp, purportedly overlaying the sensorimotor hand areas, and was stronger preceding fast compared to slow responses. This ERD response became bilateral during the 10p reward conditions before both fast and slow trials, but not during the 0p or 1p reward conditions.

Theta-band oscillations showed fronto-central synchronisation prior to the target stimulus, a response associated with increased attention and effort (Angelidis et al. 2018; Rajan et al. 2018; Basar-Eroglu et al. 1992; Klimesch 1999). This was, however, not modulated by reward or response speed, showing that it was not related motor preparation or may have a ceiling effect.

The alpha-band ERD in posterior parietal regions is likely to refer to the activation of regions involving visual-spatial coordination localised in the posterior parietal cortex (Ibos and Freedman 2016; Whitlock 2017; Assmus et al. 2005; Corbetta et al. 2000). ERD in posterior parietal electrodes has also been observed during the preparation of shoulder movements (Stancak et al. 2000). This may indicate more generalised motor readiness during intense effort, which may, initially, involve larger muscle groups even if the target movement is only a hand movement. The alpha-band ERD in the prefrontal regions supports the hypothesis that this region is implicated in motor preparation, or in the activation of cortical areas involved in motor preparation (e.g., motor areas or the basal ganglia) (Aron and Poldrack 2006). This interpretation is strengthened by the significant correlation between alpha-band ERD and individual RTs, and the present results show that these effects can be elicited by increasing performance-based rewards.

Fast compared to slow motor responses were preceded by increased beta-ERD in electrodes overlying the contralateral sensorimotor cortex, which is likely to refer to increased motor preparation during fast trials (Ishii et al. 2019; Tzagarakis et al. 2015; Fry et al. 2016; Tewarie et al. 2018). The effect of reward on beta-band oscillations is supported by previous research, in which voluntary movements have been shown to be preceded by ERD in bilateral sensorimotor cortical regions (Little et al. 2018; Stancak et al. 1997; Stancak and Pfurtscheller 1996a; Neuper and Pfurtscheller 2001; Fry et al. 2016). A similar effect was found by Stancak et al. (1997), where desynchronization in the beta band manifested in the ipsilateral somatosensory region under intermediate, but not zero, external load. The results of the present study adds to the literature by showing that incentive can elicit this effect, possibly relating to a ceiling effect in the contralateral sensorimotor cortex, boosting motor readiness in the ipsilateral sensorimotor cortex under strong effort.

Overall, the results of experiment 2 show increases in cortical activation in parietal and central electrodes paralleling increases in reward and shortening of RTs. These associations between amplitude decreases of cortical oscillations, and reward and performance could relate to the heightened level of motor readiness assumed to underlie fast responses in the horse-race theory motor control (Logan and Cowan 1984).

## General discussion

The present results add weight to our current understanding of cognitive effort by suggesting that reward may modulate effort through the activation or inhibition of relevant cortical areas in the short epoch preceding a speeded motor response in a sustained vigilance task. However, results suggest that the cortical mechanisms employed differ widely depending on the structure of the vigilance task.

If the task was conducted as a series of speedy movements executed under the same reward level (experiment 1) a sustained motor preparation was required which lasted throughout the entire block. Optimal motor performance was likely achieved as a combination of high motor readiness and inhibition in the frontal cortex, where the inhibitory component, indexed as increases of beta-band oscillations in frontal electrodes, prevailed.

In contrast, if the experiment was conducted with the three reward conditions alternating in a pseudo-random fashion with cues signalling the reward levels at the start of each trial (experiment 2), optimal performance could be achieved by a continuous build-up of activation in task-relevant cortical regions. This version of the sustained vigilance task allowed the cortical regions to reach a resting state after each movement because participants were certain that no motor response was required in the time period preceding the reward cue stimulus. Thus, to achieve a fast response, the activation in the sensorimotor, premotor and other cortical areas would need to increase from a state of low activation and reach a state of high activation within the span of two to three seconds. This process of building activation in the sensorimotor cortex did not require a parallel inhibition like in experiment 1, in which short RTs would be achieved if the sensorimotor cortex was continuously active.

A novel result was found in experiment 2, showing that when participants are offered sufficient reward (10p) activations are found bilaterally in the sensorimotor cortex. This indicates that sufficiently strong motivation can lead to motor preparation being employed in both the contralateral and ipsilateral motor areas, and adds to previous research finding bilateral sensorimotor ERD during movement (Little et al. 2018; Stancak and Pfurtscheller 1996a; Stancak et al. 1997; Neuper and Pfurtscheller 2001; Fry et al. 2016). This suggests that this effect occurs due to activation from the contralateral region ‘spilling-out’ into, or employing resources from the ipsilateral region. Movement-related ERD has been found to be stronger and more bilateral in elderly compared to younger participants (Derambure et al. 1993; Vallesi et al. 2010). The present results suggest this effect occurs because elderly participants have to make more of an effort to make the same movement compared to younger participants.

Taken together, the cortical oscillatory patterns seen in experiments 1 and 2 act according to the horse-race model (Logan et al. 1984). The horse-race model assumes two antagonised processes, one generating a response to the primary task and the other inhibiting it. In experiment 1, the increases of beta-band power in frontal cortical regions preceding fast responses in the high-reward condition could be the manifestation of the inhibition process. This would be expected to be found in the frontal cortex, which has been shown to mediate motor inhibition in stop-signal and go/no-go tasks (Wessel and Aron 2015; Aron 2007; Sakagami et al. 2006), perhaps via the subthalamic nucleus in the basal ganglia (Fischer et al. 2017; Aron 2007; Eagle and Robbins, 2003). This may also relate to an optimization of dopamine levels in the prefrontal cortex, which has been associated with increased cognitive stability (Sharp et al. 2016; Cools 2016; Cools et al. 2002; Durstewitz et al. 2000), and may, therefore, be required in experiment 1 due to the block design. In experiment 2, the time courses of ERD in the alpha and beta band showed a build-up during the interval preceding the motor response (Fig. 6a, b). This was motivationally relevant and occurred in areas associated with motor preparation and visuo-spatial attention (Fry et al. 2016; Tewarie et al. 2018; Ibos and Freedman 2016; Whitlock 2017), possibly showing the excitatory components posited by the horse-race theory.

The individual value of effort did not correlate with either amplitude increases in beta-band oscillations in experiment 1, or beta-band decreases in experiment 2. It is likely that individual values of effort are implemented during the decision about whether to engage into an effortful cognitive task, but not during an ongoing task. Expected reward level, on the other hand, acted as a modifier of effort by imposing a top-down modulation of the inhibitory and excitatory processes to boost performance. Our results also add weight to the idea of cognitive effort being the result of cognitive control (Shenhav et al. 2013; Kurzban 2016), a signal which modulates the task-appropriate inhibition and excitation of cortical response. This ties into to the horse-race model of motor control and shows that these responses can be modulated by monetary incentives. However, while a significant correlation was found between RTs and oscillatory changes between fast and slow responses, no significant relationship was found between the effects of incentives on oscillatory changes and the effect of incentives on RTs, meaning that it is difficult to directly infer that incentives altered behaviour through oscillatory changes. This may be due to other factors modulating how incentives affected RTs, such as individual or state differences, or due to a low level of statistical power.

## Conclusion

Decreasing RTs as the result of the presence and magnitude of reward was associated with cortical oscillatory changes in both experiment 1 and experiment 2. Experiment 1 showed a modulation of response-speeds on cortical inhibition in frontal, prefrontal, and central regions, especially under high reward, suggesting that high reward modulated RTs through the holding and release of inhibition. Experiment 2 showed a modulation of cortical activation over motor, frontal and posterior-parietal regions, suggesting that reward modulated RTs through changes in motor preparation and visuo-spatial co-ordination in this modified task. Taken together, these results show the dual-processes proposed by the horse-race model of motor action, showing that both inhibition and preparation can be manipulated using performance-based rewards, and ties these to the hypothesis that cognitive effort results from top-down cognitive control, and can be encouraged with monetary incentives.
